# SZBC-AI4TCM: a comprehensive web-based computing platform for traditional Chinese medicine research and development

**DOI:** 10.3389/fphar.2025.1698202

**Published:** 2025-11-17

**Authors:** Jidong Lang, Kaimin Guo, Jinna Yang, Pengcheng Yang, Yu Wei, Jingwen Han, Shuang Zhao, Zhihong Liu, Haowei Yi, Xin Yan, Binbin Chen, Cheng Wang, Jian Xu, Jiawei Ge, Wen Zhang, Xuezhong Zhou, Jiansong Fang, Jing Su, Kaijing Yan, Yunhui Hu, Wenjia Wang

**Affiliations:** 1 Tianjin Tasly Digital Intelligence Chinese Medicine Technology Co., Ltd., Tianjin, China; 2 Wecomput Technology Co., Ltd., Beijing, China; 3 Department of Artificial Intelligence, Beijing Key Laboratory of Traffic Data Mining and Embodied Intelligence, School of Computer Science & Technology, Beijing Jiaotong University, Beijing, China; 4 State Key Laboratory of Traditional Chinese Medicine Syndrome, Science and Technology Innovation Center, Guangzhou University of Chinese Medicine, Guangzhou, China

**Keywords:** traditional Chinese medicine, artificial intelligence, deep learning, bioinformatics, web-based computing platform, Alzheimer’s disease

## Abstract

**Introduction:**

In recent years, the increasing complexity and volume of data in traditional Chinese medicine (TCM) research have rendered the conventional experimental methods inadequate for modern TCM development. The analysis of intricate TCM data demands proficiency in multiple programming languages, artificial intelligence (AI) techniques, and bioinformatics, posing significant challenges for researchers lacking such expertise. Thus, there is an urgent need to develop user-friendly software tools that encompass various aspects of TCM data analysis.

**Methods:**

We developed a comprehensive web-based computing platform, SZBC-AI4TCM, a comprehensive web-based computing platform for traditional Chinese medicine that embodies the “ShuZhiBenCao” (Digital Herbal) concept through artificial intelligence, designed to accelerate TCM research and reduce costs by integrating advanced AI algorithms and bioinformatics tools.

**Results:**

Leveraging machine learning, deep learning, and big data analytics, the platform enables end-to-end analysis, from TCM formulation and mechanism elucidation to drug screening. Featuring an intuitive visual interface and hardware–software acceleration, SZBC-AI4TCM allows researchers without computational backgrounds to conduct comprehensive and accurate analyses efficiently. By using the TCM research in Alzheimer’s disease as an example, we showcase its functionalities, operational methods, and analytical capabilities.

**Discussion:**

SZBC-AI4TCM not only provides robust computational support for TCM research but also significantly enhances efficiency and reduces costs. It offers novel approaches for studying complex TCM systems, thereby advancing the modernization of TCM. As interdisciplinary collaboration and cloud computing continue to evolve, SZBC-AI4TCM is poised to play a strong role in TCM research and foster its growth in addition to contributing to global health. SZBC-AI4TCM is publicly for access at https://ai.tasly.com/ui/\#/frontend/login.

## 1 Introduction

Traditional Chinese medicine (TCM) is a valued aspect of Chinese heritage, with a long history and widespread use. It encompasses substances and approaches used for the prevention, diagnosis, and treatment of diseases, as well as rehabilitation and health maintenance. The substances are derived primarily from natural sources, such as plants, animals, minerals, and some chemical and biological products, with plant-derived products being predominant. Its pharmacological theories, such as “four natures and five flavors”, “ascending-descending-floating-sinking”, “meridian tropism”, “toxicity”, “compatibility”, and “contraindications”, are applied in clinical practice through the “syndrome differentiation and treatment” approach. Unlike Western medicine, which often targets single component or pathway, TCM operates through a multi-components, multi-target paradigm, which presents unique challenges in research, such as complex compositions, unclear mechanisms, and quality control issues. Although traditional experimental methods have contributed to the development of TCM, their time and resource requirements can be prohibitive.

Recent advances in bioinformatics, computational biology, and artificial intelligence (AI) have opened new avenues for TCM research. These techniques can improve efficiency and success rates across the entire research and drug development pipelines, from initial discovery to clinical trials (Zhang et al., 2024; Wu et al., 2024a; Zhang et al., 2022; Li and Zhang, 2023; Chu et al., 2020; Song et al., 2024). For example, AlphaFold3 can accurately predict the 3D structures of biological molecules (e.g., proteins, DNA, and RNA) and their interactions, offering immense potential for disease research and drug delivery innovation (Abramson et al., 2024). The AutoDock suite enables efficient virtual screening of molecular docking and can facilitate structure-based drug design within approximately 5 hours (Forli et al., 2016; Eberhardt et al., 2021; Trott and Olson, 2010). Song et al.’s compositional message passing neural network predicts the absorption, distribution, metabolism, excretion, and toxicity properties of molecules, which can increase drug development success rates while reduce costs (Song et al., 2020).

Despite these advancements, computational applications in TCM face significant hurdles. First, the complexity and diversity of TCM data pose challenges related to data collection and standardization. Second, the generalizability of AI models and bioinformatics tools in the TCM context is often constrained by the unique characteristics of TCM data, a factor that has spurred the proliferation of specialized tools. These challenges demand significant efforts from researchers to gather and deploy resources, including high-performance GPUs and other costly hardware. Finally, many tools are difficult to deploy and lack user-friendliness due to their reliance on advanced programming and server maintenance skills, which further impedes the efficiency and modernization of TCM research.

To address these challenges, we developed SZBC-AI4TCM, a comprehensive web-based platform that integrates cutting-edge AI algorithms and bioinformatics tools to streamline TCM research. The platform combines a user-friendly and interactive visualization framework with hardware acceleration and leverages server and high-performance computing resources to expedite data analysis. Designed for accessibility, it caters particularly to wet-lab researchers and those without programming expertise. To demonstrate its utility, we present the TCM research related to Alzheimer’s disease (AD) as an example, showcasing its capabilities in data mining, drug screening, and mechanism analysis based on network pharmacology and molecular docking.

## 2 Materials and methods

### 2.1 Web-based framework design

**FIGURE 1:**
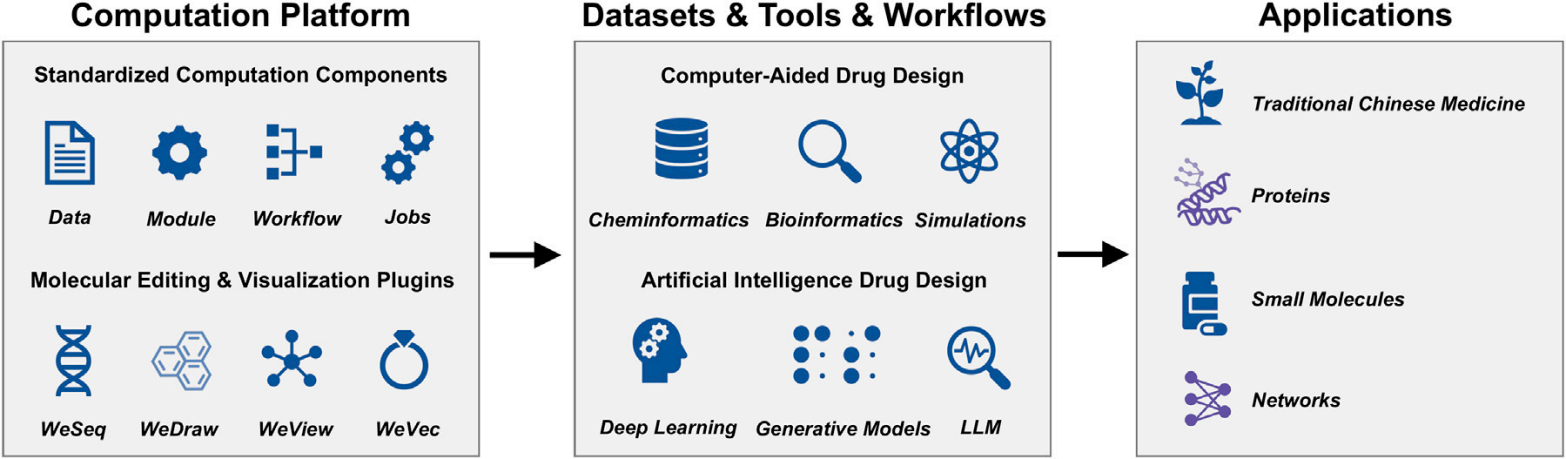
Schematic of the WeMol platform framework.

The platform utilizes the WeMol computational framework (https://wemol.wecomput.com), developed by Wecomput Technology Co., Ltd., to manage and maintain the analytical modules. WeMol incorporates state-of-the-art streaming architecture, data standardizing capability, modules, workflows, and tasks into its computational processes. This allows users to efficiently manage data, AI tools, workflows, and computational jobs (Figure 1). The framework includes integrated plugins for molecule editing and visualization, such as WeDraw (small molecule editing), WeView (molecular structure visualization), WeSeq (macromolecular sequence editing), and WeVec (gene sequence editing).

### 2.2 SZBC-AI4TCM platform and features

**FIGURE 2:**
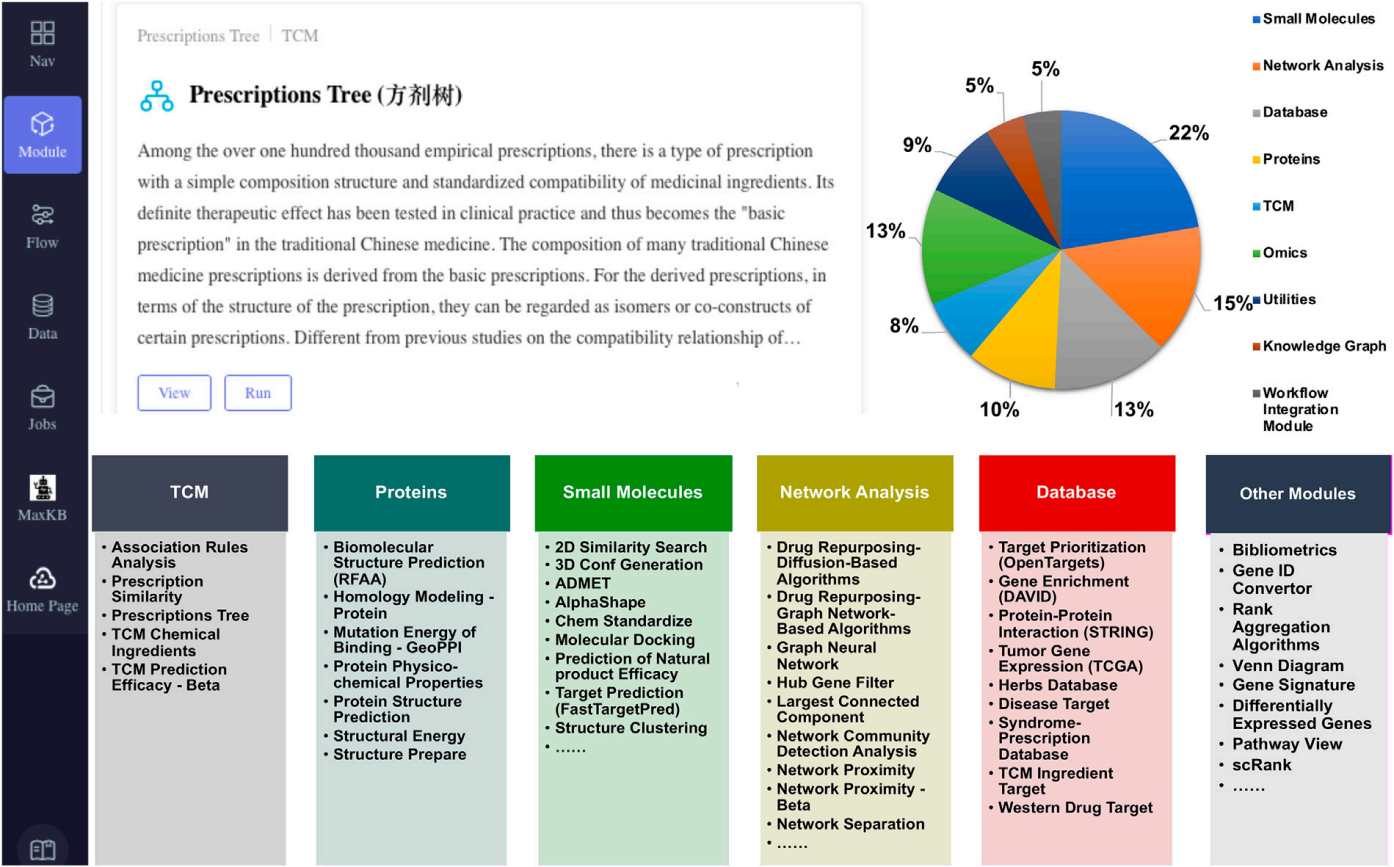
Overview of the SZBC-AI4TCM platform. Upper part: User interface and proportional distribution by functional group; Lower part: List of the modules within each main group.

As Figure 2 shows, SZBC-AI4TCM has a web-based user interface, which allows users to use it easily and interactively. The platform currently comprises 67 analytical modules, mainly categorized into five functional groups: TCM analysis (5 modules), protein analysis (7 modules), small-molecule analysis (15 modules), network analysis (10 modules), and databases (9 modules). Other functions are scattered or still in development (total of 21 such modules). These modules relate to TCM formulation analysis, mechanism elucidation, and drug screening (Figure 2; Supplementary Table S1). Three example workflows are provided: Network Pharmacology Analysis Workflow, Network Framework for Drug Re-purposing Workflow, and Knowledge Graph Analysis Workflow. The platform is hosted on a Dell PowerEdge R730XD server featuring an Intel(R) Xeon(R) CPU E5-2673 v4 @ 2.30 GHz (80 threads), 250 GB of memory, and an NVIDIA GeForce RTX 2080 Ti GPU. The operating system is CentOS Linux 8. As an illustration, the Network Pharmacology Analysis Workflow requires approximately 6 min to process a query for “diabetes” with the listed botanical drugs (salvia miltiorrhiza, panax notoginseng and borneol in chinese input), delivering the core network gene set, enrichment analysis, and visualization. Similarly, the Molecular Docking module completes an analysis of a receptor (1STP.pdb) against 100 ligands (demo_100_3D.sdf) under a rigid model in roughly 8 min. This performance markedly enhances analytical throughput and accelerates research development. Users can customize workflows but may need additional modules for integration. For intelligent querying, the platform incorporates Max Knowledge Base (https://github.com/1Panel-dev/MaxKB), an open-source quality assurance system based on large language models (LLMs) and retrieval-augmented generation powered by the Qwen-72B LLM. This feature offers real-time support, helping users resolve queries and understand tools/methods with minimal learning effort. Due to computational and security constraints, this feature is currently limited to intranet access.

## 3 Results

### 3.1 Statistical depiction and analysis of TCM formulations

The SZBC-AI4TCM platform provides system analysis modules related to TCM, including retrieval of disease-related formulations from the TCM prescription database, analysis of the association rules of formulations, and analysis of formulations similarity. On this platform, we named the prescription database as the “Syndrome-Prescription Database”, which contains a total of 3,716 formulations. To illustrate our research and the application of the platform’s modules, we take Alzheimer’s disease (AD) as an example. First, we conducted a search using “Alzheimer’s disease” as the keyword. This search retrieved 399 AD-related formulations (Supplementary Tables S1,S2), all of which represent potential therapeutic or interventional strategies for AD.

**FIGURE 3:**
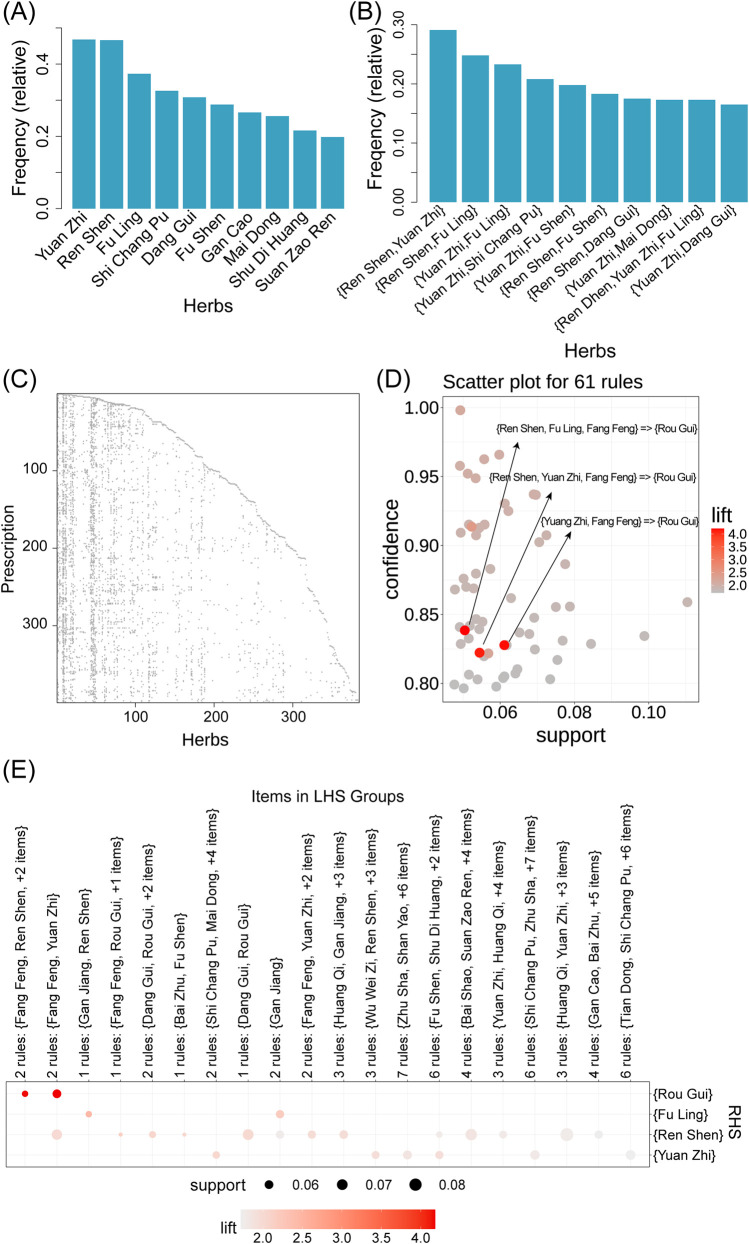
Analysis of 399 formulations related to Alzheimer’s disease using the SZBC-AI4TCM platform. **(A)** Frequency of simple botanical drug. **(B)** Frequency of combination of two or more botanical drugs. **(C)** Scatter plot showing the distribution of botanical drugs in the formulations. **(D)** Scatter plot for 61 association rules. **(E)** Visualization of items in the left-hand side groups of the association rules. The size of the dot represents support value, and the color represents the lift valuer.

Using the “Association Rules Analysis” module, we performed a frequency analysis of the individual botanical drugs and botanical drug combinations mentioned in the 399 retrieved formulations (Supplementary Tables S1,S2). The results revealed the top 10 most frequently used botanical drugs (Figure 3A), including Yuan Zhi (*Polygalae Radix*), Ren Shen (*Ginseng Radix Et Rhizoma*), Fu Ling (*Poria*), Shi Chang Pu (*Acori Tatarinowii Rhizoma*), Dang Gui (*Angelicae Sinensis Radix*), Fu Shen (*Poria cum radix pini.*), Gan Cao (*Glycyrrhizae Radix Et Rhizoma*), Mai Dong (*Ophiopogonis Radix*), Shu Di Huang (*Rehmanniae Radix Praeparata*), and Suan Zao Ren (*Ziziphi Spinosae Semen*). Numerous studies have reported the therapeutic potential of the aforementioned botanical drugs for AD. For example, in Liu et al.’s study, Polygalae Radix (Yuan Zhi) was shown to alleviate cognitive decline in AD mouse models by mitigating β -amyloid toxicity and targeting the extracellular signal-regulated kinase pathway (Li et al., 2024). Multiple therapeutic mechanisms of ginseng-derived ginsenosides against AD have been reported. For example, Rb1 modulates synaptic plasticity, reduces inflammation, and inhibits apoptosis; Rb targets the tau protein in APP transgenic mice; and Rg1 demonstrates anti-apoptotic and antioxidant effects in APP/PS1 mice (Mook-Jung et al., 2001; Wang et al., 2018; Yang et al., 2020; Zhang et al., 2021; Li et al., 2021). Other botanical drugs, such as Fu Ling, Shi Chang Pu, Dang Gui, Shu Di Huang, and Suan Zao Ren have also been extensively studied for AD treatment (Chen et al., 2013; Song et al., 2018; Trott and Olson, 2010; Wang et al., 2022; Su et al., 2023).

The frequency analysis of botanical drugs revealed synergistic relationships between botanical drug pairs. Notably, Ren Shen and Yuan Zhi co-occurred in 116 of 399 (29%) formulations, and Ren Shen with Fu Ling (25%) and Yuan Zhi with Fu Ling (23%) also exhibited high co-occurrence frequencies (Figure 3B; Supplementary Tables S1,S2). Using the “Association Rules Analysis” module that employs the apriori algorithm, we systematically mined botanical drug combinations from the 399 formulations associated with AD (Figure 3C) (Hahsler et al., 2005). With the parameter settings of support threshold = 0.05 and confidence threshold = 0.8, 61 statistically significant botanical drug association rules were identified (Figures 3D,E; Supplementary Tables S2,S3). In the Apriori algorithm, lift quantifies the enhancement effect of the antecedent occurrence on the consequent occurrence. A lift value >1 indicates that the antecedent increases the likelihood of the consequent; =1 implies independence between them; and <1 suggests the antecedent reduces the consequent’s probability (Hahsler et al., 2005). After sorting the rules in descending order by the lift value, the association rules “{Ren Shen, Fu Ling, Fang Feng (*Saposhnikoviae Radix*) => Rou Gui}“, “{Yuan Zhi, Fang Feng => Rou Gui (*Cinnamomi Cortex*)}“, and “{Ren Shen, Yuan Zhi, Fang Feng => Rou Gui}” had the lift values of 4.21, 4.18, and 4.12, respectively (i.e., all substantially exceeding 1; refer to Figures 3D,E). This demonstrates that the co-occurrence of the botanical drug combinations, “Ren Shen - Fu Ling - Fang Feng”, “Yuan Zhi - Fang Feng” or “Ren Shen - Yuan Zhi - Fang Feng” significantly increased the probability of Rou Gui appearing consequently. All three rules have high confidence levels (>0.8) . These specific combinations may exert synergistic therapeutic effects on AD-related cognitive improvement, which provides insights into the formulation principles of traditional herbal formulations.

For formulation similarity analysis, pairwise comparisons of the 399 AD-related formulations were conducted using the “Formula Similarity” module (Supplementary Tables S2–S4). The results demonstrated that some formulation pairs exhibited high similarity in botanical drug composition, suggesting they may originate from the same theoretical system (e.g., TCM syndrome differentiation principles) or shared clinical empirical knowledge, with potential common therapeutic mechanisms against AD.

Based on the association rules analysis results (Supplementary Tables S2,S3), we ranked the 61 filtered botanical drug combinations by their support value and selected seven key botanical drugs from the top four rules for AD targeted formulation screening. These botanical drugs were Fu Ling, Rou Gui, Ren Shen, Fang Feng, Yuan Zhi, Mai Dong, and Shu Di Huang. All of these had high-frequency occurrence and robust association rules. Therefore, formulations containing all seven botanical drugs were hypothesized to possess good therapeutic efficacy. Among the 399 formulations, only three contain all these seven botanical drugs (Table 1). The taxonomic and medicinal details of botanical drugs in the three formulations are detailed in Supplementary Tables S2–S5. One of them is a formulation named “Shuyu Wan”, which has been previously associated with AD treatment (Zhou, 2022; Cheng et al., 2021; Ma et al., 2022; Qiu, 2020). In the subsequent sections, we use the Shuyu Wan as a representative example of a formulation to demonstrate our platform’s functional modules.

**TABLE 1:** Details of three formulations selected based on the botanical drug frequency and association rules.

Indication	Formulation name	Composition
Alzheimer’s disease	Baifuling Sanfang	Fu Ling, Yuan Zhi, Zhi Gan Cao, Rou Gui, Ren Shen, Bai Shao, Fang Feng, Shu Di Huang, Tie Fen, Huang Qi, Mai Dong
Alzheimer’s disease	Changpu Wan	Shi Chang Pu, Du Zhong, Shu Di Huang, Fu Ling, Ren Shen, Dan Shen, Fang Feng, Bai Zi Ren, Bai Bu, Yuan Zhi, Wu Wei Zi, Shan Yao, Mai Dong, Rou Gui
Alzheimer’s disease	Shuyu Wan	Shan Yao, Yuan Zhi, Shu Di Huang, Tian Dong, Fu Shen, Long Chi, Di Gu Pi, Fang Feng, Fu Ling, Mai Dong, Ren Shen, Rou Gui, Wu Wei Zi, Che Qian Zi

### 3.2 Comprehensive analysis at the disease level

For disease-related analysis, the SZBC-AI4TCM platform offers a suite of powerful analytical modules to facilitate in-depth exploration of the disease mechanisms. Using AD as a case study, we demonstrate the integrated application of five core modules: 1) “Disease Target”; 2) “Protein–Protein Interaction (STRING)”; 3) “Hub Genes Identification”; 4) “Largest Connected Component”; and 5) “Gene Enrichment (DAVID)”.

#### 3.2.1 Disease Target module

This module enables rapid screening of disease associated genes across multiple integrated databases. The platform consolidates data from DisGeNET (Piñero et al., 2016), eDGAR (Babbi et al., 2017), GWAS (Hindorff et al., 2009), Pharos (Kelleher et al., 2022), MalaCards (Rappaport et al., 2013), and 23 sub-databases under the Open Targets Platform (Koscielny et al., 2016). We queried it with the keyword “Alzheimer’s disease”. For this study, we selected the DisGeNET database as a representative source. After filtering the entries without valid gene IDs, 3,384 AD-related genes were identified (Supplementary Tables S1–S3), including well-established pathogenic genes such as APP, PSEN1, and PSEN2. This module can substantially increase researchers’ efficiency in aggregating disease–gene associations from heterogeneous databases.

#### 3.2.2 Protein–protein interaction (STRING) module

This module integrates the functional components from the STRING database to enable direct protein–protein interaction (PPI) network analysis of disease related genes (Mering et al., 2003). Inputting the 3,384 AD-related genes into this module with a confidence threshold set to 0.9 (i.e., retaining only the highest confidence interactions) yielded 11,268 PPI pairs involving 2,778 disease-associated genes (Supplementary Tables S2,S3).

#### 3.2.3 Hub Genes Identification module

Hub genes in a disease gene network play pivotal roles in determining the modular characteristics of the network. In a disease gene network, hub genes tend to be involved in regulating biological processes or pathological states. The identification of hub genes aims to pinpoint key regulators within complex gene networks that critically influence biological functions or disease progression, thus providing essential insights into pathogenesis, therapeutic target discovery, and drug development.

**FIGURE 4:**
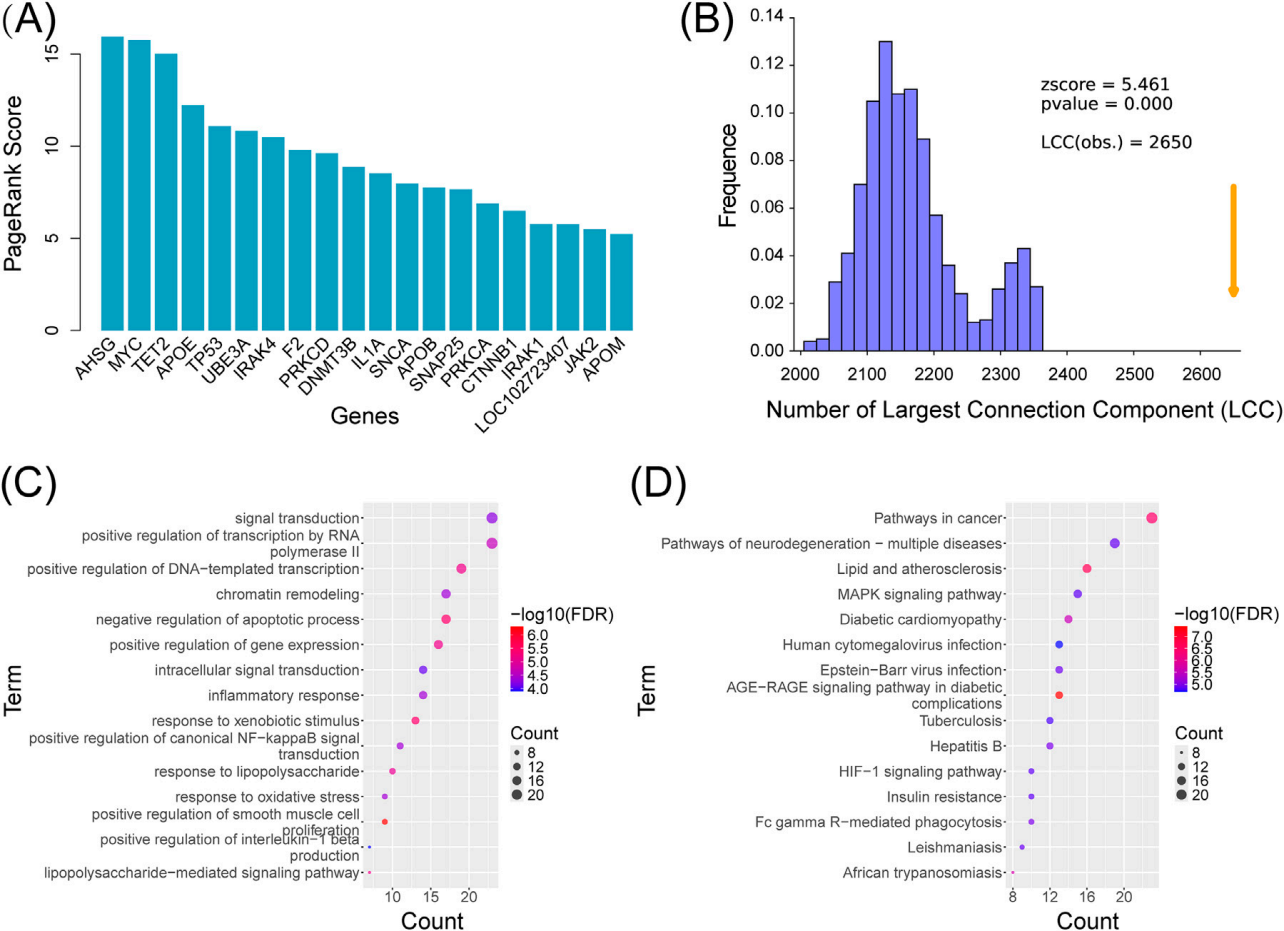
Analysis of Alzheimer’s disease–associated genes using the SZBC-AI4TCM platform. **(A)** Top 20 prioritized genes associated with Alzheimer’s disease by the PageRank algorithm deployed on the platform. **(B)** Analysis of the largest connected components for genes associated with Alzheimer’s disease. **(C)** GO enrichment analysis. **(D)** KEGG enrichment analysis.

The SZBC-AI4TCM platform employs the PageRank algorithm for hub gene prioritization (Page et al., 1999). This algorithm assigns importance scores to genes based on their topological positions within the PPI network, where in higher scores indicate greater functional significance and network centrality. The AD-associated PPI network comprised 2,778 gene nodes and 11,268 interactions (Supplementary Tables S2,S3). This module generated quantified the score for each node. Subsequently, ranking the genes by descending scores enabled systematic identification of disease-relevant hub genes (Supplementary Tables S3–3). The top 20 highest-scoring genes are visualized in Figure 4A.

#### 3.2.4 Largest connected component module

Numerous studies indicate that disease-associated genes are not randomly distributed in PPI networks but tend to form interconnected sub networks, existing as cohesive “communities” within the global network (Goh et al., 2007). Largest connected component (LCC) analysis identifies the largest and most densely interconnected sub network (“community”) in disease gene networks. This approach enables the extraction of the maximally connected disease sub-network while facilitating, through comparison with randomly sampled networks, an accuracy assessment of the identified disease genes-ones that can represent the disease to a certain extent. Using the SZBC-AI4TCM platform, we performed LCC analysis on the 2,778 AD-related genes. The resultant LCC contained 2,650 genes with 32,686 interactions (Supplementary Tables S3,S4). Comparative analysis with 1,000 randomly sampled networks (matched in gene number) demonstrated that AD-associated genes exhibited significantly higher connectivity (permutation test, Zscore=5.461 ) and formed a cohesive disease-network (Figure 4B).

#### 3.2.5 Gene Enrichment (DAVID) module

This module enables Gene Ontology (GO) and Kyoto Encyclopedia of Genes and Genomes (KEGG) pathway analyses of disease-associated genes. Using the top 100 AD related hub genes prioritized by the PageRank scores (Supplementary Tables S2,S3), we performed functional enrichment analysis to identify the significantly overrepresented biological processes and pathways. The GO analysis revealed enrichment in critical AD-related processes, including inflammatory response (GO:0006954, $FDR=4.56\times 10^{-5}$ ), response to oxidative stress (GO:0006979, $FDR=4.56\times 10^{-5}$ ), and positive regulation of canonical NF- κ B Signaling (GO:0043123, $FDR=4.56\times 10^{-5}$ ) (Figure 4C). KEGG pathway analysis highlighted pathways of neuro-degeneration (KEGG: hsa05022, $FDR=1.44\times 10^{-5}$ ), MAPK signaling (KEGG: hsa04010, $FDR=1.49\times 10^{-5}$ ), and AGE-RAGE signaling pathway in diabetic complications (KEGG: hsa04933, $FDR=3.71\times 10^{-8}$ ) as key mechanisms (Figure 4D). These results indicated the modular functional architecture of AD-associated hub genes and their convergence on core pathological pathways.

### 3.3 Network pharmacology and molecular docking

The SZBC-AI4TCM platform enables systematic network pharmacology analysis relating to TCM through the following workflow: 1) Herbal metabolite extraction (“TCM Ingredient Target” module); 2) Target retrieval (“TCM Ingredient Target” module); 3) Target–gene comparative analysis (“Venn Diagram” module); 4) Network proximity analysis (“Network Proximity” module); 5) Molecular docking (“Molecular Docking” module). Using the formulation, Shuyu Wan, which is an AD targeting formulation identified in the previous analysis as a representative case, we demonstrate the integrated application of this analytical framework.

**FIGURE 5:**
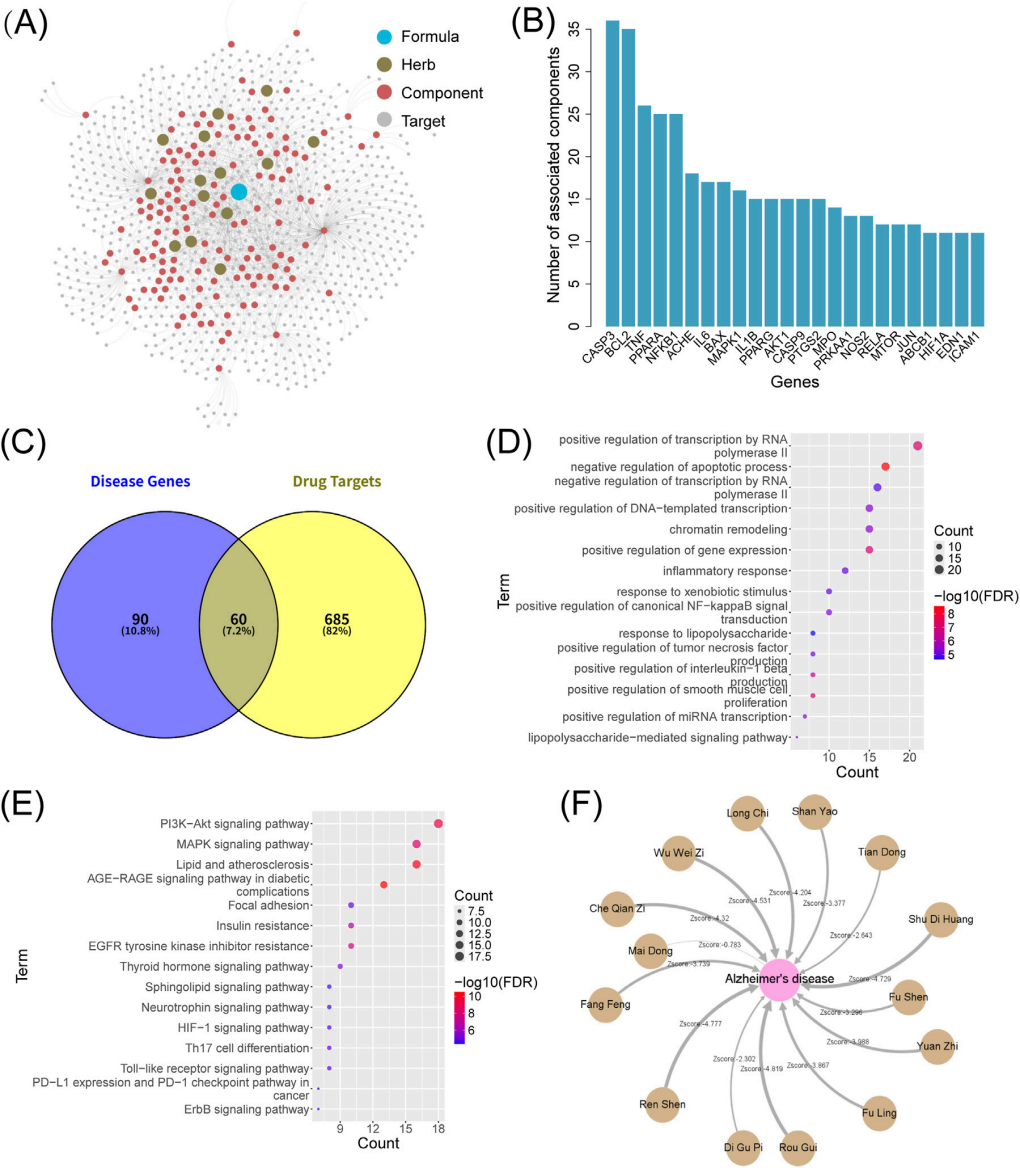
Network pharmacology analysis for the formulation Shuyu Wan using the SZBC-AI4TCM platform. **(A)** Network of formulation–botanical drugs–metabolites–targets. **(B)** Number of metabolites associated with targets (only showing targets with number of metabolites more than 10). **(C)** Venn diagram between the genes associated with AD (the top 150 prioritized genes) and the formulation’s targets. **(D)** GO enrichment analysis, and **(E)** KEGG enrichment analysis of the overlapping genes between the AD gene set and drug target gene set. **(F)** Network proximity analysis between botanical drugs in the formulation and AD. All Zscores are less than 0, and the weight of the edges in network represents the magnitude of the Zscore.

Using the “TCM Ingredient Target” module of the SZBC-AI4TCM platform, we analyzed data from the HIT2 database. After filtering out the entries lacking valid gene IDs, 143 bioactive metabolites derived from 14 botanical drugs in Shuyu Wan were mapped to 2,083 genes, averaging approximately 14 genes per metabolite (Figure 5A; Supplementary Tables S1–S4). Removing duplication yielded 745 unique genes, indicating that multiple metabolites might target same genes. The CASP3 gene exhibited the highest metabolite association (36 metabolites), followed by 14 genes each linked to >15 metabolites (Figure 5B; Supplementary Tables S1–S4). For drug–disease gene comparison, the top 150 AD-related hub genes (ranked by PageRank scores in Supplementary Tables S3–3) were selected as the AD gene set. Venn diagram analysis using the “Venn Diagram” module revealed 60 overlapping genes between the 745 drug target gene set and the AD gene set (Figure 5C; Supplementary Tables S2–S4). Functional enrichment analysis via the “Gene Enrichment (DAVID)” module demonstrated that these shared genes were significantly enriched in GO terms related to transcriptional regulation, apoptotic process, and inflammatory response (Figure 5D). KEGG pathway analysis highlighted key AD associated pathways, such as the PI3K-Akt signaling pathway (KEGG: hsa04151, $FDR=6.16\times 10^{-10}$ ), MAPK signaling pathway (KEGG:hsa04010, $FDR=3.41\times 10^{-9}$ ), thyroid hormone signaling pathway (KEGG: hsa04919, $FDR=3.28\times 10^{-6}$ ), sphingo lipid signaling pathway (KEGG: hsa04071, $FDR=2.45\times 10^{-5}$ ), and neuro-trophin signaling pathway (KEGG: hsa04722, $FDR=2.26\times 10^{-5}$ ) (Figure 5E). These pathways exhibited significant associations with AD pathogenesis, particularly in neuronal survival and neuro-inflammation regulation.

Using the “Network Proximity” module, we systematically assessed the therapeutic potential of Shuyu Wan against AD at both the botanical drug and metabolite levels. In the botanical drug-level analysis, target sets for each botanical drug were constructed by aggregating the non-redundant targets and all their metabolites. Network proximity analysis between the 14 botanical drugs and the AD gene set revealed that all botanical drugs exhibited negative Zscores. Lower values of the Zscore may indicate stronger therapeutic relevance. Notably, 11 out of 14 botanical drugs had Zscore <−3 , indicating strong AD-targeting efficacy (Figure 5F; Supplementary Tables S3–S4). In the analysis of herbal metabolites, out of 143 metabolites, 79 had a Zscore of <−1 , 54 had a Zscore of <−2 , and 21 had a Zscore of <−3 . (Supplementary Tables S4–4). These findings robustly validate Shuyu Wan’s multi-scale therapeutic effects against AD through synergistic interactions among botanical drugs and metabolites.

**FIGURE 6:**
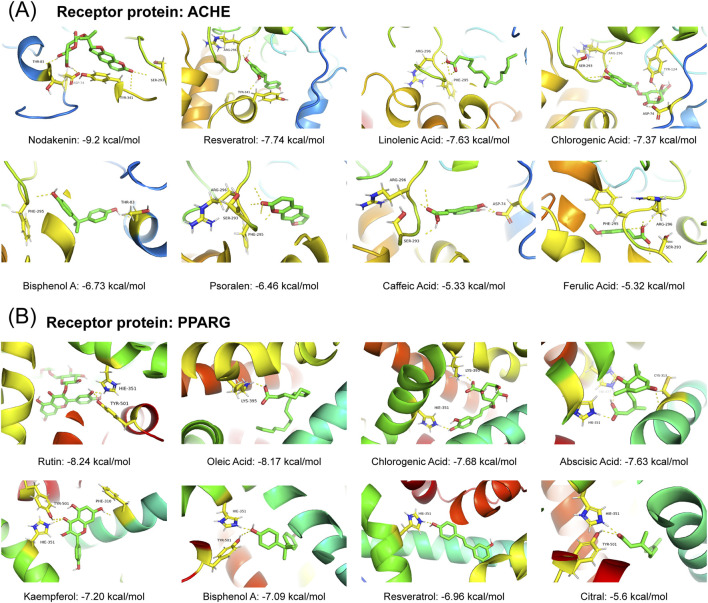
Visualization of the molecular docking of different ligands with the corresponding receptor protein. **(A)** ACHE as the receptor protein. **(B)** PPARG as the receptor protein. The corresponding score indicates the binding energy (kcal/mol).

We also validated bioactive metabolites using the platform’s functionality. We employed the “Molecular Docking” module to predict binding modes and interactions and obtained binding energy and affinity data. Target selection was performed based on the metabolite–target list (Supplementary Tables S1–S4), where 24 targets associated with more than 10 metabolites were prioritized, including ACHE (acetyl-cholinesterase) and PPARG (peroxisome proliferator activated receptor gamma) (Supplementary Tables S4–S5). ACHE was linked to 15 metabolites: chlorogenic acid, ethanol, bisphenol A, caffeic acid, ginsenoside Rg1, α -linolenic acid, resveratrol, vitexin, cinnamic acid, ferulic acid, forsythiaside, gallic acid, ursolic acid, nodakenin, and psoralen. PPARG interacted with 12 metabolites: chlorogenic acid, citral, rutin, ethanol, bisphenol A, kaempferol, mannose, oleic acid, resveratrol, vanillin, abscisic acid, and ursolic acid. Five metabolites were shared between both targets. These metabolites were employed as ligand molecules and subjected to molecular docking against their respective receptor proteins (ACHE and PPARG). In this process, the SDF files of ligands (except ginsenoside Rg1) were retrieved from PubChem and processed (merging and hydrogenation) using Open Babel (v3.1.1) (O’Boyle et al., 2011). Crystal structures of ACHE (PDB:6O4W) and PPARG (PDB: 6FZG) were obtained from the RCSB PDB database (https://www.rcsb.org/). Non-essential molecules and water were removed, followed by hydrogenation with the PyMOL software (Schrödinger and DeLano, 2020). Rigid docking was performed using AutoDock deployed on the SZBCAI4TCM platform, with the binding pockets defined by co-crystallized ligands. All ligand–receptor pairs exhibited binding energies <−2 kcal/mol, except urs olic acid with PPARG (1.28 kcal/mol), indicating strong binding capabilities (Supplementary Tables S4–S6). Notably, 57% of the ACHE-ligand complexes and 67% of the PPARG-ligand complexes showed energies <−5 kcal/mol (Figure 6). These results demonstrated strong binding capacity and enhanced molecular interactions between the analyzed receptor proteins and ligand molecules while also validating the accuracy of our target identification for the metabolites within the TCM formulation.

### 3.4 Drug screening

**FIGURE 7:**
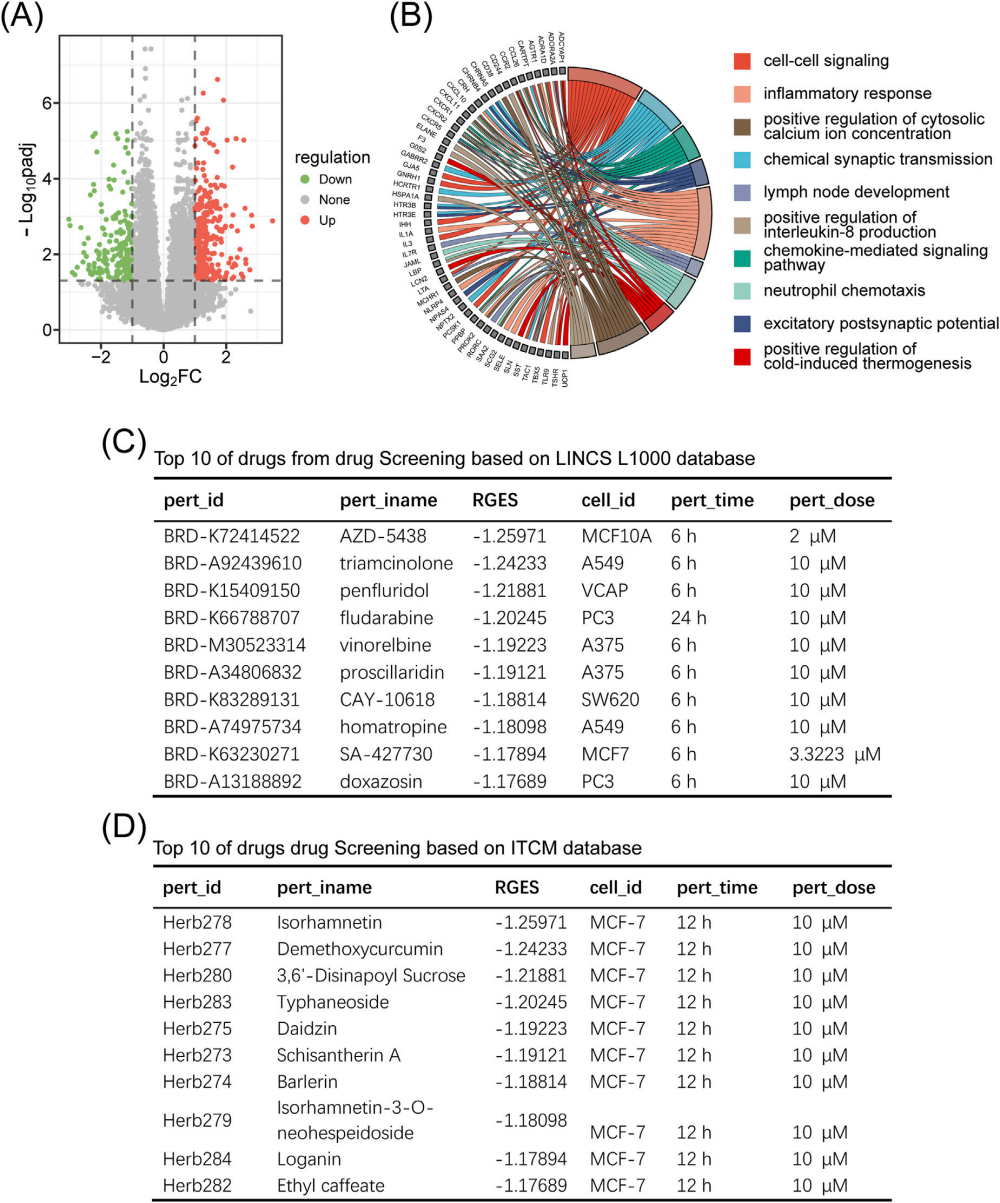
Drug screening using the SZBC-AI4TCM platform. **(A)** Volcano plot showing gene expression changes between cases and controlsfor Alzheimer’s disease (GSE159699). **(B)** GO enrichment analysis of the differential expression genes. **(C)** Top 10 drugs from the drug screening based on the LINCS L1000 database. **(D)** Top 10 drugs from the drug screening based on the ITCM database.

We retrieved RNA-seq raw count data (GSE159699) from the Gene Expression Omnibus (GEO) database, comprising 30 temporal lobe samples (12 AD cases and 18 controls) (Supplementary Tables S1–S5) (Nativio et al., 2020). Using the “Differentially Expressed Genes” module with the thresholds of FDR<0.05 and |log2FC|>1 , we identified 459 AD associated differentially expressed genes (284 upregulated and 175 downregulated) (Figure 7A; Supplementary Tables S2–S5). Functional enrichment analysis via the Gene Enrichment (DAVID) revealed significant GO terms related to AD pathogenesis, including cell–cell signaling (GO:0007267, $pvalue=5.13\times 10^{-7}$ ), neutrophil chemotaxis (GO:0030593, $pvalue=3.23\times 10^{-5}$ ), inflammatory response (GO:0006954, pvalue=0.002 ), and chemical synaptic transmission (GO:0007268, pvalue=0.002 ) (Figure 7B; Supplementary Tables S3–S5).

The “Gene Signature” module was employed to identify potential therapeutics for AD using the LINCS (Library of Integrated Network based Cellular Signatures) L1000 and ITCM (Integrated Traditional Chinese Medicine) databases (Supplementary Tables S4–S5). This module calculates a disease-reversion score by quantifying the ability of compounds to reverse disease-specific gene expression patterns. Negative scores indicate reversal of AD-associated expression (therapeutic potential), and positive score suggest synergy with disease mechanisms (therapeutic risk). The top 10 ranked small molecules (LINCS) and natural products (ITCM) are shown in Figures 7C,D. Notably, six out of the top 10 LINCS small molecules (including penfluridol, fludarabine, vinorelbine, and doxazosin) have been previously reported to exhibit anti-AD effects in peer-reviewed studies (Nativio et al., 2020; Chiba et al., 1997; Lehrer and Rheinstein, 2017; Shamsi et al., 2024; Rahman et al., 2020; Rathi et al., 2025), corroborating the validity of our screening approach.

## 4 Discussion

Traditional Chinese Medicine (TCM) constitutes a precious cultural heritage of the Chinese nation, embodying millennia of accumulated clinical theories and practical experience in disease prevention and treatment. It has long demonstrated unique advantages in disease prevention and treatment, and it is garnering increasing attention from the global scientific community in recent years. The remarkable advancements in AI and bioinformatics have exerted profound and transformative impacts on TCM research and development. The digitization of biomedical research and intelligent computing have become robust trends in the field (Zhou et al., 2024; Xu, 2024; Buller et al., 2025). However, several challenges persist in TCM research, such as: 1) The inherently complex nature of TCM data, posing difficulties in collection and standardization; 2) Limited generalizability of the existing computational tools/methods for drug development; and 3) Technical barriers in tool deployment and utilization. These factors have significantly constrained the progress of TCM research.

To address these challenges, we developed SZBC-AI4TCM, a comprehensive web-based computational platform that integrates state-of-the-art AI algorithms, bioinformatics tools, and TCM databases. This one-stop solution provides robust computational support, enabling researchers to obtain analytical results more efficiently and thereby enhancing the productivity and success rate of TCM research. The platform supports multilingual inputs for specific analysis modules. For example, the TCM Targets Search BATMAN module accepts inputs in Pinyin, Chinese, English, and Latin. However, other modules, such as the Herbs Database, currently only support Chinese. This is because many underlying TCM databases are primarily in Chinese, and integrating comprehensive multilingual support requires substantial translation and curation efforts. We acknowledge that this limitation can impair the user experience and potentially affect analytical accuracy, and therefore, its resolution is a key focus for our next development phase. Meanwhile, We have noted that some TCM names (including formulas, medicinal materials and ingredients) often exhibit significant variation across realworld data sources. For example, the same entity might appear as “Shuyu Pill”, “Shuyu Wan”, or “Shu Yu Wan”. Such inconsistencies stem from multiple factors, including differences in Pinyin transliteration, word segmentation conventions, and database design. This lack of standardization can lead to incomplete information retrieval and compromise analytical accuracy. While our current platform does not include dedicated modules to address these variations, we are actively developing a comprehensive TCM synonym dictionary. Furthermore, we plan to leverage large language model (LLM)-based Retrieval-Augmented Generation (RAG) technology for automated name normalization and are designing standardized preprocessing workflows specifically for TCM texts to significantly enhance data consistency and analytical precision in future work.

Using the TCM research in Alzheimer’s disease as an example, we demonstrated the platform’s capabilities in multiple research domains, including TCM formulation data mining, drug screening, mechanism analysis based on network pharmacology analysis and molecular docking. This platform significantly reduces reliance on traditional trial-and-error approaches, while also drastically lowering the time, labor, and financial costs associated with the development of TCM. In addition, based on the modules of this platform, we have also applied them in other studies. For instance, relying on the network proximity module, Yang et al. conducted an analysis of network proximity between vascular calcification associated genes and the targets of Compound Danshen Dripping Pills (CDDP) in their research on the treatment of vascular calcification using this drug (Yang et al., 2024). This analysis was used to evaluate the potential therapeutic effect of the drug on vascular calcification. Meanwhile, Wang et al. applied network proximity to identify the potential pathological mechanisms of Alzheimer’s disease (AD) associated with YangXue QingNao Wan (YXQNW) by integrating the drug-target network (Wang et al., 2024). In the study by Zhao et al., based on the Bibliometrics module on the SZBC-AI4TCM platform, the research focused on exploring the research hotspots and trends in Tourette Syndrome (TS), as well as the roles and potential mechanisms of the botanical drug pairs related to Shaoma Zhijing Granules and their main metabolites in the treatment of TS (Zhao et al., 2024). This work laid a foundation for analyzing the therapeutic mechanism of Shaoma Zhijing Granules in TS and provided evidence support for its clinical application.

SZBCA-I4TCM features a user-friendly web interface with intuitive operation. Integrated with the MaxKB question answering system, the platform facilitates rapid comprehension of each analytical module’s operational procedures and underlying principles, which considerably lowers the learning curve for researchers. Moreover, the analytical modules and workflow in the platform will be regularly optimized and iteratively upgraded. The development and deployment of cutting-edge technical modules, particularly the “Knowledge Graph” module group and the “Large Language Model Application” module group, will substantially enhance the platform’s technical support capabilities. The integration of the existing innovative technical modules not only provides users with a systematic and professional toolkit for TCM research but also offers unique methodological value in critical research scenarios, such as drug interaction analysis and prescription compatibility pattern mining. Of particular note is the platform’s independently developed “Prescriptions Tree” module (Lang et al., 2025), which employs phylogenetic tree construction algorithms to propose innovative solutions for research directions in TCM formulations and TCM formulations’ classification. Through the ongoing development of such analytical tools, a distinctive methodological framework addressing key scientific questions in TCM research will gradually take shape, offering new technical pathways to overcome industry research bottlenecks. However, some limitations of SZBC-AI4TCM warrant acknowledgment: 1) The quality and completeness of the foundational data require continuous updates and supplementation. 2) The currently implemented tools and methodologies may not encompass all computational requirements, necessitating periodic expansion, updates, and optimization of the analytical modules. 3) The intelligent Q&A functionality remains under development and would benefit from integration with additional LLMs, such as ChatGPT (Vaswani et al., 2023) and DeepSeek-AI et al. (2025a); Wu et al., 2024b; DeepSeek-AI et al., 2025b). 4) While designed with user friend lines in mind, the platform’s advanced features still present a non-trivial learning curve that may require targeted training for certain user groups. 5) Exponential growth in computational resource demands is anticipated with increasing module deployment and user adoption, mandating systematic resource scaling.

It is worth noting that although the databases integrated into the platform were initialized with the most recent versions available at the time of development, they are not currently synchronized in real-time with their official sources due to the substantial resource and cost implications involved. The primary objective of the first phase of the SZBC-AI4TCM project is to establish a comprehensive suite of analytical functions for traditional Chinese medicine research. To this end, we have localized several publicly available databases and tools to support users in conducting various analyses. Currently, the platform does not perform in-depth integration or comprehensive evaluation of the results generated by these tools/databases, and each analysis is performed independently. Therefore, the outputs should be regarded as preliminary references, and we strongly reiterate and encourage users to perform further evaluation and experimental validation, such as *in vitro* assays, animal models, or clinical trials, to remain essential to confirm their biological relevance (Magalhães et al., 2021; Bolz et al., 2021). A key part of our ongoing development strategy includes periodic updates to these underlying databases. Our current plan is to perform updates on a quarterly basis, or more frequently based on significant user demand and newly available data.

In summary, SZBC-AI4TCM represents a significant milestone in the integration of TCM with modern computational technologies. By providing a comprehensive, scalable, and accessible platform for TCM research, we anticipate this tool will substantially enhance the efficiency and effectiveness of TCM-based drug discovery and development. Future efforts will focus on three key directions: 1) Enhancing the platform through functional upgrades; 2) Expanding the analytical tools, algorithms, and databases; and 3) Fostering global collaboration within the TCM research community to advance the modernization and internationalization of TCM.

## 5 Conclusion

SZBC-AI4TCM is a comprehensive web-based computational platform specifically designed for TCM research and development. The platform integrates an extensive collection of cutting-edge AI algorithms, bioinformatics tools, and specialized TCM databases, collectively offering robust computational solutions that can significantly reduce research costs and dramatically enhance development efficiency from a computational perspective. We envision that SZBC-AI4TCM will serve as a powerful computational backbone for both TCM research and clinical applications. Its continued development and implementation are expected to make substantial contributions to the advancement, modernization, and globalization of TCM.

## Data Availability

Publicly available datasets were analyzed in this study. This data can be found here: (Gene Expression Omnibus (GEO): GSE159699; PubChem database: https://pubchem.ncbi.nlm.nih.gov/; LINCS data portal: https://lincsportal.ccs.miami.edu/dcic-portal/; ITCM database: http://itcm.biotcm.net/). Further inquiries can be directed to the corresponding authors.
